# STARS: Spread the beauty of DNA Crystallography

**DOI:** 10.1063/4.0001034

**Published:** 2025-10-27

**Authors:** Chenyi Andrew, Susanna Huang

**Affiliations:** Georgia Institute of Technology, Atlanta, Georgia, USA

## Abstract

Very few freshmen at Georgia Tech have heard about DNA or Protein crystallography, not to mention its importance in structural advancement of protein functions, or let alone its beauty in nature. From salts to proteins to crystals and ultimately to DNA, crystallography is an elegant technique that provides insightful visions in the details of the protein structure through diffraction data and modeling. Crystallography is not only inherently beautiful, forming protein crystals from nucleation of protein and nucleic acid molecules, but can aid in the development of targeted therapeutic treatments through crystal structures. The world of crystallography is fascinating, and the Georgia Tech branch of STARS crystallography research club aims to show students of all levels in Atlanta the beauty and importance of DNA and protein crystallography.

STARS offers students both practical experience and valuable insights into the world of crystallography through engaging lectures, research lab tours, and hands-on wet-lab crystallization experiments. At Georgia Tech, undergraduate research opportunities can be limited and often difficult to access—especially for early undergraduates. STARS addresses this gap by hosting crystallography workshops open to the entire Georgia Tech community, providing students of all backgrounds the chance to engage with and gain experience in research. Beyond the college campus, STARS also has an outreach committee that focuses on introducing the wonders of crystallography to students from K-12. In the past, STARS has organized programs and activities that engage students with the idea of crystallography and crystal-growing skills. For example, STARS has hosted crystal-growing competitions for K-12th students to come together, interact, and put their science knowledge skills into use. From these programs, the students are not only drawn into the world of crystallography but also are able to acquire new lab skills such as micropipetting, note-taking, and data analysis.

As a first-year freshman passionate about DNA crystallization at Georgia Tech, STARS stood out to me, not only because it's a club that focuses on crystallography but also its uniqueness in providing hands-on lab experience on campus. After joining the club, I was fascinated with the number of experiences and skills that STARS offered. One of the first activities I participated in involved growing lysozyme crystals and optimizing the crystallization conditions to achieve large, single-crystal growth. From the experiment, I was able to gain practice in setting up the experiment and the experimental iterations of note-taking to assess the quality of crystal growth and adjusting conditions for growth optimization in response.

Now, as an active member of STARS, I hope to contribute to its mission by helping expand access to the world of crystallography—especially for students and aspiring young scientists who are still discovering and shaping their passions for the future.

